# Patient Experiences With Nirmatrelvir/Ritonavir for COVID-19 in a Collaborative Care Model: A Cross-Sectional Study on Self-Management, Information, and Medication Impact

**DOI:** 10.1177/23743735251342126

**Published:** 2025-05-14

**Authors:** Beatriz Santos, Maeva Rousseau, Ralitza Gauthier, Alexandra Calmy, Marie P. Schneider

**Affiliations:** 1 Institute of Pharmaceutical Sciences of Western Switzerland University of Geneva, University of Lausanne, Geneva, Switzerland; 2 School of Pharmaceutical Sciences, 27212 University of Geneva, Geneva, Switzerland; 3 Pharma24, Academic Community Pharmacy, Geneva, Switzerland; 4 HIV/AIDS Unit, Infectious Diseases Division, Geneva University Hospitals, Geneva, Switzerland

**Keywords:** physician–pharmacist collaboration, nirmatrelvir/ritonavir, COVID-19, long-term medication, medication adherence, patient experience, interprofessional collaboration

## Abstract

In response to the COVID-19 pandemic, nirmatrelvir/ritonavir was approved as the first per os treatment to prevent severe disease progression. This study explores patients’ self-management of nirmatrelvir/r and its impact on their long-term medications, and their experience of the information provided by pharmacists and physicians. Adults receiving nirmatrelvir/r from September 2022 to March 2023 were interviewed via a semistructured telephone survey. Sociodemographic, clinical data, and experience with nirmatrelvir/r and cotreatments were collected and analyzed descriptively. Of the 281 patients receiving nirmatrelvir/r, 100 (36%) participated in this study. Ninety (90%) adhered to nirmatrelvir/r, while 5 (5%) reported early discontinuation and 5 (5%) increased time between dosages. Information gaps regarding side effects, risks, benefits, and mechanisms of action were identified. Due to interactions with nirmatrelvir/r, 43% (n = 43/85) of cotreatments were temporarily interrupted, and 13% (n = 11/85) of doses were adjusted. Patient management of nirmatrelvir/r and cotreatments was successful, but satisfaction toward nirmatrelvir/r information could be improved. This study highlights the importance of ongoing efforts to ensure communication strategies, patient education, and interprofessional collaboration in providing treatments with drug–drug interactions.

## Introduction

In June 2022, in response to the COVID-19 pandemic, the Swiss Agency for Therapeutic Products (Swissmedic) granted temporary authorization for the use of nirmatrelvir boosted with ritonavir–nirmatrelvir/r as the first early and oral treatment for COVID-19 infection to prevent progression to severe disease.^1^ Nirmatrelvir/r was previously granted Emergency Use Authorization by the Food and Drug Administration in December 2021, as well as conditional authorization by the European Medicines Agency in January 2022.^2,3^

Nirmatrelvir is a protease inhibitor that targets all human-infecting coronaviruses, while ritonavir is a well-known potent inhibitor of cytochromes P450 (CYP) 3A4 and 2D6, and the Pgp transporter which enhances nirmatrelvir's activity.^4,5^ According to the Swiss Society for Infectious Diseases guidelines, nirmatrelvir/r is recommended within 5 days of symptom onset for individuals aged 12 years and older with confirmed infection, particularly those at moderate to high risk of developing a severe form of the disease, including the elderly, patients with multiple long-term conditions (polymorbidity), or immunocompromised.^6–8^ Polymorbid patients, defined as individuals with 2 or more long-term conditions,^9^ are frequently prescribed multiple long-term medications^10^ and are at an increased risk of drug interactions, adverse effects, and complex treatment management.^11,12^

Although nirmatrelvir/r is an effective treatment against COVID-19, it has safety limitations because of its clinically significant pharmacokinetic interactions with commonly prescribed treatments for long-term diseases that are metabolized by cytochromes P450, such as cardiovascular therapies, neuropsychiatric agents, immunosuppressants, and others.^13,14^ For example, ritonavir significantly increases the concentrations of simvastatin, a lipid-lowering drug metabolized exclusively by CYP3A4, and this combination is therefore contraindicated.^15^ Some patients may therefore need to temporarily suspend or modify their long-term treatments during and after the end of the nirmatrelvir/r therapy until ritonavir has been eliminated (5 half-lives, 40 h).^4^ The recommended regimen is 300 mg nirmatrelvir (two 150 mg tablets) and 100 mg ritonavir (one 100 mg tablet) taken every 12 h for 5 days, which may be subject to adaptations, particularly in case of moderate to severe kidney failure. Prescribing and dispensing nirmatrelvir/r requires a thorough medication review (including over-the-counter and alternative medicine) and evaluation in close interprofessional collaborative practice. Collaboration with the patient is required to verify their understanding and ensure patient safety.^4^

In Geneva, the delivery of nirmatrelvir/r in the outpatient setting was set up collaboratively between the local health authorities, the University Hospitals (infectious disease specialists, emergency physicians, and clinical pharmacists), and the academic outpatient pharmacy at the exit of the University Hospitals. During the first semester of marketing (July-December 2022), most nirmatrelvir/r treatments were dispensed by this academic outpatient pharmacy, and after December 21, 2022, every pharmacy was allowed to dispense.

While research has primarily focused on the effectiveness and safety of nirmatrelvir/r in hospital and ambulatory settings,^16,17^ there is limited understanding of patients’ self-management of nirmatrelvir/r therapy, particularly in outpatient contexts. Limited attention has been given to patients’ experiences with the information provided by healthcare professionals, including pharmacists and physicians, and the impact of nirmatrelvir/r on the management of their ongoing long-term cotreatments. Addressing these gaps is critical to ensure that nirmatrelvir/r therapy is integrated safely and effectively into existing treatment regimens, supported by structured interprofessional collaboration among physicians, pharmacists, and patients. This study aims to address these gaps by evaluating the following: (1) patients’ self-management of nirmatrelvir/r, (2) patients’ experience of information provided by pharmacists and physicians on nirmatrelvir/r and patients’ cotreatments, and (3) patients’ reported the impact of nirmatrelvir/r on their usual long-term cotreatments.

## Methods

This observational cross-sectional survey was conducted through a telephone-based interview from September 2022 to March 2023.

### Setting and Training of Pharmacists

This study was conducted in the academic pharmacy located at the exit of the University Hospitals in Geneva, Switzerland. A French e-learning codesigned locally by physicians and pharmacists was provided to all pharmacists dispensing nirmatrelvir/r. The main topics were: (1) nirmatrelvir/r indication and regimen, (2) contraindications, (3) dosage adaptations related to specific health conditions such as renal or liver insufficiency, (4) drug–drug interactions and their management through the COVID-19 drug interaction checker of the University of Liverpool,^18^ and (5) main side effects. When dispensing nirmatrelvir/r, pharmacists must complete a checklist to ensure that any potential interactions or contraindications have been addressed, and that information has been provided to the patient (see Supplemental Data). The pharmacist also ensured that the patient started the treatment within 5 days of symptoms and informed the patient on how to take the treatment using the tablet pack as a memory aid. The patient was advised not to start any other medication during the treatment and for 3 days after without the advice of a healthcare professional.^13^

### Recruitment and Data Collection

This study included adults who had been prescribed nirmatrelvir/r for COVID-19 infection. We excluded individuals who could not provide consent due to cognitive impairment and could not answer the interview in French or English. A routine follow-up call was conducted by a community pharmacist 5 to 7 days after the treatment was dispensed in the pharmacy. At the end of the follow-up call, patients were asked if they agreed to be contacted by a University researcher by phone within 3 to 5 days to answer questions about the nirmatrelvir/r provision and use based on an anonymous survey. We aimed to recruit 100 consecutive participants through convenience sampling during an acute period of the COVID-19 epidemic (September-December 2022) without knowing whether nirmatrelvir/r would be widely prescribed in outpatient practice. When contacted by the researcher, participants were informed that they could withdraw from the study until the end of the interview and that they could interrupt the interview without justification. Participants were informed that the data collected was strictly confidential, neither their pharmacist nor their physician would be informed of their data, which was analyzed anonymously for research purposes only.

### Study Variables and Measurements

We developed an 11-item multiple choice and Likert scale survey in French divided into 5 main themes: (1) Sociodemographic data such asage, gender, medication anamnesis (all currently prescribed medications and over-the-counter [OTC] medications) and self-rated general health on a 5-point Likert scale (very poor to very good) at 2 time points (recalling the time just before starting nirmatrelvir/r and at the time of the interview) (1 item), (2) Characteristics of nirmatrelvir/r dispensing process such as the type of prescriber (eg, general physician, specialist), and dispensing either to the patient or a third party (2 items), (3) Patient self-management of nirmatrelvir/r (how nirmatrelvir/r was taken and if any adjustments were made to the initial recommended prescription) (2 items), (4) Perceived quality and quantity of information on nirmatrelvir/r and usual long-term cotreatments on a 10-point Likert scale (4 items), and (5) The impact of nirmatrelvir/r on the use of long-term medications during the 5 days of treatment and the 3 days following the end of treatment with nirmatrelvir/r (2 items). The outcomes and respective endpoints of this study, measured at 8 to 10 days after treatment dispensing, are as follows: (1) The types of self-adjustments made by patients to their prescribed nirmatrelvir/r regimen, compared to the original prescription and dispensation (eg, treatment discontinuation, dose skipping, change in dosing schedule). (2) The impact of the nirmatrelvir/r prescription on patients’ self-management of their long-term comedications, including temporary interruptions, not resumed treatments and changes in medication, (3) Patients’ evaluations and perceptions of the quality and quantity of information provided by their physicians and pharmacists regarding nirmatrelvir/r and their long-term comedications assessed through a 10-point Likert scale.

The survey (Supplemental Material) was pretested by 3 patients with long-term conditions before the start of the study to identify and address issues with the clarity and relevance of the questions. No modifications were made after this point.

### Data Analysis

Long-term diseases were categorized according to the *International Classification of Diseases* code,^19^ and medication were coded using the Anatomical Therapeutic Chemical Classification system (ATC codes).^20^ We conducted descriptive analysis on the collected variables and outcomes (mean and standard deviation, median, and interquartile range [IQR] as appropriate).

To assess differences in participants’ perceptions of their general state of health before and after nirmatrelvir/r, as well as differences in perceptions of the information provided by pharmacists and physicians, we conducted a Wilcoxon signed-rank test since the data consisted of paired measurements and did not meet the normality assumption. A Mann-Whitney *U* test was performed to compare information ratings between individuals who collected the treatment themselves and those who did not. A *P* value of less than .05 was considered statistically significant. Statistical analysis was performed using STATA 17.0.

### Ethics

Our protocol was submitted to the **Geneva ethics committee on research involving humans –(Protocol Nr 2022-01364)**, who responded that, in our study, “improving the quality of the service” clearly predominates over the “research” aspect and did not need to be formally approved by the ethics committee. We obtained a waiver; no written informed consent was required, and the data were collected anonymously.

## Results

Among 281 individuals who received nirmatrelvir/r between September 2022 and March 2023, 165 (59%) routine follow-up calls were conducted, which resulted in 100 individuals participating in the telephone interview. Half of the participants collected nirmatrelvir/r between November and December 2022. The flowchart of patients’ inclusion can be found as Supplemental Data. All individuals were prescribed nirmatrelvir/r according to the criteria of the Swiss Society for Infectious Diseases except for 3 individuals who got it to prevent dissemination to close frail relatives they oversaw.

Participants in this study had a median age of 64 years old (IQR: 53.5-77.0) and 53% were female. Perceived general health improved over the 7-day course with nirmatrelvir/r (*P* < .001), as shown in Table 1. Eighty-five participants (85%) reported taking at least one long-term medication at the time of the study, with a median of 3 medications per participant (IQR: 1.0-5.0), mainly for the cardiovascular system (24%), alimentary tract and metabolism (22%), and blood-forming organs such as antithrombotic medications (11%) (see Table 1). Thirty-four percent of reported medications have been taken for 10 years or more.

**Table 1.** Sociodemographic Data, Prescribed Long-Term Medications, and Perceived General Health.

		N (%)
**Age range (years) (n = 100; min = 26, max = 90)**		
18-40		10 (10)
41-60		34 (34)
61-80		39 (39)
≥81		17 (17)
**Gender (n = 100)**		
Female		53 (53)
**Health-related data**		N (%)
Medication (ATC Code) (n = 338 reported by n = 85 participants)		
Cardiovascular system		84 (25)
Alimentary tract and metabolism		76 (22)
Blood and blood-forming organs		39 (12)
Nervous system		29 (9)
Antineoplastic and immunomodulating agents		28 (8)
Systemic hormonal preparations (excl. sex hormones and insulin)		22 (7)
Others^a^		22 (7)
Respiratory system		21 (6)
Anti-infectives for systemic use		17 (5)
**Time since the start of each long-term medication (n = 338 reported by n = 85 participants)**		
10 years or more		114 (34)
5-9 years		75 (22)
2-4 years		67 (20)
1 year or less		30 (9)
Unsure or unable to answer		52 (15)
**Perceived general health (n = 100)**		
	At the start of nirmatrelvir/r (*as recalled by participant during the interview*), n (%)	At the time of the interview, n (%)
Very good	8 (8)	15 (15)
Good	38 (38)	46 (46)
Average	26 (26)	27 (27)
Poor	22 (22)	11 (11)
Very poor	6 (6)	1 (1)

^a^ Others include genitourinary system and sex hormones, musculoskeletal system, antiparasitic products, sensory organs, and various.

## Participants Management of Nirmatrelvir/r

Fifty-five percent (n = 55) of participants were prescribed nirmatrelvir/r by a general physician, and 71% (n = 71) of participants collected the treatment themselves at the pharmacy, as presented in Table 2.

**Table 2.** Prescription and Delivery, and Nirmatrelvir/r Adjustments by Healthcare Professionals and Participants.

				N (%)
**Prescriber**				
General physician				55 (55)
Specialist (outpatient)				33 (33)
Hospital service (inpatient)				6 (6)
Emergency department				6 (6)
**Nirmatrelvir/r was collected at the pharmacy by … (n = 100)**				
Patient				71 (71)
Family member				23 (23)
Friend or third party				4 (4)
In-home nurse				2 (2)
**Participants whose usual regimen was adjusted by physician or pharmacist (n = 10/100)**		**Participants having a prescription with the usual regimen (nirmatrelvir 300 mg with ritonavir 100 mg twice daily for 5 days) (n = 90/100)**		
**Participants having self-adjusted nirmatrelvir/r without professional recommendation and reasons (n = 10/90)**					
150 mg Nirmatrelvir + 100 mg Ritonavir every 12 h for 5 days	5 (50)		Treatment discontinuation after 24-48 h (n = 5)	> 12 h between doses (n = 5)		
300 mg Nirmatrelvir + 100 mg Ritonavir every 12 h for 7 days	2 (20)	Side effects	5	2 (40)
		Omissions/forgetting	/	3 (60)
300 mg Nirmatrelvir + 100 mg Ritonavir every 12 h for 4 + ½ days	1 (10)			
300 mg Nirmatrelvir + 100 mg Ritonavir every 12 h for 3 days	1 (10)			
300 mg Nirmatrelvir + 100 mg Ritonavir every 12 h for 1-2 days	1 (10)			

Nirmatrelvir/r was prescribed with a dosage of 300 mg nirmatrelvir with 100 mg ritonavir orally twice daily for 5 days to 90 participants (90%), while it was prescribed or dispensed differently, respectively, by a pharmacist or a physician in 10 (10%) individuals. For 5 participants, the dosage of nirmatrelvir/r was reduced by half (150 mg twice a day), the official recommended dosage in case of moderate to severe renal failure. For 5 other participants, the duration of treatment was reduced to 1 to 2 days instead of 5.

Ten out of 90 participants who had been prescribed 300 mg nirmatrelvir with 100 mg ritonavir orally twice daily for 5 days self-adjusted their prescription. Five (50%) increased the time between 2 doses by more than 12 h and 5 (50%) others reduced the length of the treatment by 1 or 2 days. Among these 10 participants, 7 reported adjusting the treatment due to side effects (nausea, diarrhea, stomach pain), while 3 reported having forgotten one or more doses, which led to an increase in time between doses. Only one of these 10 participants discussed and confirmed the adaptation with a healthcare professional.

## Completeness of Information Received

Among the 85 participants taking at least one long-term medication, 94% (n = 80/85) reported complete satisfaction (10/10) regarding information received on the management of their long-term medication during the nirmatrelvir/r treatment, while 55% (n = 55/100) did so regarding nirmatrelvir/r. Individuals who collected nirmatrelvir/r themselves rated information quantity slightly higher than those who relied on a third party, though this difference was not statistically significant (*P* = .65). Missing information reported for nirmatrelvir/r was about side effects (n = 22), potential risks (n = 15), and benefits (n = 13) of the medication, as reported in Table 3. Some individuals reported missing information regarding their long-term medications, such as interactions with nirmatrelvir/r (n = 3) or how to manage cotreatments (whether to temporarily stop (n = 1) or to continue taking them during treatment with nirmatrelvir/r (n = 3). Some participants reported filling in the missing information using other sources, particularly the internet or social media (n = 20) (see Table 3). Finally, participants rated the quality of information provided by physicians and pharmacists as high, with a slightly but not significantly higher rating for pharmacists (*P* = .07) (see figure as Supplemental Data).

**Table 3.** Types of Reported Missing Information Regarding Nirmatrelvir/r and Long-Term Cotreatments.

		N (%)
Side effects		22 (49)
Potential risks of the medication		15 (33)
Benefices of the medication		13 (29)
Medication mechanism of action		10 (22)
Interactions with other medications		4 (9)
Dosage		1 (2)
Schedule (when to take it)		1 (2)
**Missing information regarding long-term medications (n = 5 participants among 85 participants with long-term medications)**		N (%)
Interactions with nirmatrelvir/r		3 (60)
How to continue the usual medications during nirmatrelvir/r?		3 (60)
Whether to temporarily stop usual medication during nirmatrelvir/r		1 (20)
**Alternative sources of information consulted by participants for seeking missing information**		
	For nirmatrelvir/r n (%)	For long-term medications n (%)
Internet/social media	19 (42)	1 (20)
Through further discussion with a pharmacist	4 (9)	/
Through the medication package insert	3 (7)	/
Through a family member / friend	2 (4)	/
Through further discussion with the physician	1 (2)	1 (20)

## Impact on Long-Term Medications

For 41 participants (48%), nirmatrelvir/r had a short-term impact on long-term cotreatments as these participants reported temporary interruptions, dosage increases or reductions, medication switches, and delays in intake, see Table 4.

**Table 4.** Impact of Nirmatrelvir/r on Use of Long-Term Cotreatments.

			N (%)
Stopping ≥ 1medication(s)			37 (90)
Reducing the dosage of ≥ 1medication(s)			9 (22).
Increasing the dosage of ≥ 1medication(s)			2 (5)
Switching medications			1 (2)
Delaying medication intake (by several hours (n = 1)) or days (n = 1))			2 (5)
**Adaptations recommended by (n = 41)**			
Prescribing physician			27 (66)
Pharmacist			11 (27)
On patient's self-initiative			3 (7)
**Cotreatments adjusted (n = 61) by 41 participants**			
Increased or decreased medication dosages (n = 11, 18%)		Stopped medication (n = 50, 82%)	
Cardiovascular system	4 (36)	Cardiovascular system	26 (52)
Alimentary tract and metabolism	3 (27)	Alimentary tract and metabolism	3 (6)
Systemic hormonal preparations	1 (9)	Antineoplastic/ immunomodulating agents	9 (18)
Nervous system	3 (27)	Nervous system	4 (8)
		Musculoskeletal system	3 (6)
		Systemic hormonal preparations	3 (6)
		Others (genitourinary system, anti-infectives)	2 (4)
**Duration of adjustments to long-term cotreatments (n = 41 participants)**			
≥10 days			21 (51)
6-9 days			9 (22)
5 days			9 (22)
2-3 days			2 (5)
**Resumption of long-term medication (n = 41 participants) at the time of the survey**			
Have resumed all medications			24 (58)
Have not resumed any medication			15 (37)
Have resumed some medications			2 (5)
Plan to resume long-term medication (n = 15 participants, who have not resumed any medication yet)			15 (100)
… In the following 3 days			6 (40)
… In the following 7-10 days			5 (33)
…. Do not know			4 (27)

As shown in Table 4, 93% of adjustments to long-term medications were recommended by a healthcare professional such as a physician or a pharmacist, and over 50% were conducted for 10 days or longer, while 22% were conducted for exactly 5 days (the usual duration of nirmatrelvir/r treatment). Out of the 49 medications for which an interruption was reported, 26 were for the cardiovascular system, and 19 were for statins. At the time of participation, 58% (n = 24) of patients had reinitiated their long-term medications with the usual dosage, 5% had resumed some of them, and 37% (n = 15) had not yet resumed taking them. Among participants who had not started taking their usual medication again, 4 did not know when they would begin to.

## Discussion

The results of this study provide insight into patients’ experiences associated with the dispensing and use of nirmatrelvir/r as an early treatment for COVID-19 in an outpatient setting. Although the introduction of nirmatrelvir/r for outpatients was successfully set up thanks to an interprofessional collaboration, our results also reveal important aspects to address related to the patient experience with the information received from healthcare providers, especially regarding the management of long-term medications and the impact of the nirmatrelvir/r on these usual treatments.

Our study shows high adherence to the prescribed regimen of nirmatrelvir/r, reflecting the successful implementation of the treatment protocol in the outpatient setting with a significant improvement in perceived general health being reported among participants over the 7-day course of the treatment which aligns with the intended therapeutic effect of nirmatrelvir/ritonavir.^16^ However, some individuals self-adjusted the prescribed regimen, primarily by increasing the time between doses or reducing the treatment duration mainly due to forgetfulness or side effects, as seen in other studies,^14,21,22^ rather than the patient feeling better or no longer presenting symptoms.^23^ The link between experiencing side effects and treatment discontinuation has been vastly documented in the literature for both short- and long-term medications, suggesting the importance of discussing, monitoring, and managing side effects, especially when starting a new medication.^24,25^ This underscores the need for improved patient–provider communication to address concerns, support medication adherence, and increase patient trust.^26,27^

Additionally, coordinated patient education delivered by physicians and pharmacists using standardized procedures ensures consistent and comprehensive information, reduces the risk of conflicting information, and enhances understanding. Strengthening patient involvement by actively engaging them in the decision-making process enhances trust, satisfaction, and, ultimately, medication adherence.^28,29^ Our findings show that whether patients or caregivers collected medications did not impact on perceived information sufficiency. However, medications are often collected by a third party (caregiver or other) highlighting the importance of providing them with clear and effective communication which will ultimately reach the patient.^30^

In this study, more patients expressed complete satisfaction with information related to long-term medication than with nirmatrelvir/r. Our results identified gaps of information in key areas such as side effects, potential drug–drug interactions, and the mechanism of action of nirmatrelvir/r—topics commonly overlooked as reported in other studies.^31–33^ Interestingly, insufficient information about side effects was a key reason for early medication discontinuation in our participants. We hypothesize that unawareness of side effects may result in medication nonpersistence.^25,34^ Addressing gaps in information through effective patient-centered communication, and teach-back methods, particularly at medication initiation, is crucial for informed decision-making, patient partnership, and safety.^35,36^

Our results show that the prescription of nirmatrelvir/r has a short-term impact on participants’ long-term medications, with a significant proportion of participants experiencing temporary interruptions, dosage adjustments, or medication switches, consistent with the clinically significant drug–drug interactions reported in the literature.^4,13,22,23^ Most changes were recommended by healthcare professionals, underlining the collaborative role of pharmacists and physicians in managing concurrent medications during treatment with nirmatrelvir/r. Surprisingly, more than half of medication adjustments were conducted for 10 days or more, which is longer than the officially recommended interruption (3 days after the stop of nirmatrelvir/r),^13^ showing the need to improve the pharmacological knowledge of physicians and pharmacists and help patients return to their usual treatments with confidence after nirmatrelvir/r is completed. The high rate of interruptions in cardiovascular medications, including statins, highlights the need for careful drug-interaction management when prescribing nirmatrelvir/r, especially in patients with cardiovascular conditions.^37^ A coordinated and interprofessional approach alongside structured protocols is essential to ensure that patients safely resume their treatments.

By assessing medication regimens, identifying drug–drug interactions, and providing clear information to patients on the use of nirmatrelvir/r, pharmacists work closely with the prescribers and ensure patient safety and optimized treatment outcomes. This model of collaboration can be seen as an example of the effective and safe implementation of emerging treatments in the healthcare system. Training provided to pharmacists before the start of the nirmatrelvir/r dispensing and a standardized procedure was crucial in this context. Standardized patient education protocols developed in interprofessional collaboration, as well as tools to monitor patients’ experience of medication in an outpatient care context, should be considered for future provision of medications with a high-risk profile to ensure patient safety and satisfaction.^23,38^ The strength of this study lies in the fact that it was conducted in parallel with the introduction of outpatient dispensing of nirmatrelvir/r in Geneva; it allowed the collection of patients’ first experience with the medication. However, some limitations should be acknowledged. The sample size of 100 participants, though reasonable for a convenience sampling approach, may not capture the full spectrum of patients’ experience, and participants who agreed to participate may not be representative of the broader population of individuals prescribed nirmatrelvir/r. However, systematic calls were conducted from the pharmacy to each patient, collecting nirmatrelvir/r, limiting a potential selection bias. The exclusion of individuals with cognitive impairment and those unable to communicate in French or English may have limited the representation of vulnerable populations in the study. The interview-based nature of the data collection may limit our interpretations due to the potential for desirability and recall biases. Nevertheless, the collection of patient outcomes gave us insights into patients’ perceptions and experiences with nirmatrelvir/r. To limit the desirability bias, data were collected by a researcher independent of the clinical and pharmacy teams, and patients were informed that their physician and pharmacist would not have access to the data. Although this study focuses on a specific treatment for the management of COVID-19, its findings highlight the overall importance of patient communication and interprofessional collaboration to improve the experience of patients with long-term conditions navigating through different healthcare settings. Lastly, the study's focus on a single Geneva pharmacy may limit generalizability; however, this was the sole dispensing site in the region for the first 4 months of the study.

## Conclusion

Outpatients’ experience of using nirmatrelvir/r during COVID-19 pandemic revealed the successful implementation of the treatment. However, these experiences also highlighted challenges, particularly in terms of information provision regarding nirmatrelvir/r and the nonrecovery of long-term treatments that had to be discontinued when patients were called. This underlines the need for effective communication and patient education. Supporting patients in integrating complex short-term treatments such as nirmatrelvir/r, while ensuring the management and later resetting of long-term cotreatments requires clear and consistent communication from both physicians and pharmacists. Ongoing efforts to improve communication strategies and enhance training for healthcare professionals contribute to the safe and effective use of emerging treatments in ambulatory care settings. The reinforcement of collaboration between physicians and pharmacists is an underexplored strategy to ensure and enhance patient experience in managing their medications across various healthcare settings.

## Supplemental Material

sj-docx-1-jpx-10.1177_23743735251342126 - Supplemental material for Patient Experiences With Nirmatrelvir/Ritonavir for COVID-19 in a Collaborative Care Model: A Cross-Sectional Study on Self-Management, Information, and Medication Impact Supplemental material, sj-docx-1-jpx-10.1177_23743735251342126 for Patient Experiences With Nirmatrelvir/Ritonavir for COVID-19 in a Collaborative Care Model: A Cross-Sectional Study on Self-Management, Information, and Medication Impact by Beatriz Santos, Maeva Rousseau, Ralitza Gauthier, Alexandra Calmy and Marie P. Schneider in Journal of Patient Experience

sj-docx-2-jpx-10.1177_23743735251342126 - Supplemental material for Patient Experiences With Nirmatrelvir/Ritonavir for COVID-19 in a Collaborative Care Model: A Cross-Sectional Study on Self-Management, Information, and Medication Impact Supplemental material, sj-docx-2-jpx-10.1177_23743735251342126 for Patient Experiences With Nirmatrelvir/Ritonavir for COVID-19 in a Collaborative Care Model: A Cross-Sectional Study on Self-Management, Information, and Medication Impact by Beatriz Santos, Maeva Rousseau, Ralitza Gauthier, Alexandra Calmy and Marie P. Schneider in Journal of Patient Experience

sj-docx-3-jpx-10.1177_23743735251342126 - Supplemental material for Patient Experiences With Nirmatrelvir/Ritonavir for COVID-19 in a Collaborative Care Model: A Cross-Sectional Study on Self-Management, Information, and Medication Impact Supplemental material, sj-docx-3-jpx-10.1177_23743735251342126 for Patient Experiences With Nirmatrelvir/Ritonavir for COVID-19 in a Collaborative Care Model: A Cross-Sectional Study on Self-Management, Information, and Medication Impact by Beatriz Santos, Maeva Rousseau, Ralitza Gauthier, Alexandra Calmy and Marie P. Schneider in Journal of Patient Experience

sj-docx-4-jpx-10.1177_23743735251342126 - Supplemental material for Patient Experiences With Nirmatrelvir/Ritonavir for COVID-19 in a Collaborative Care Model: A Cross-Sectional Study on Self-Management, Information, and Medication Impact Supplemental material, sj-docx-4-jpx-10.1177_23743735251342126 for Patient Experiences With Nirmatrelvir/Ritonavir for COVID-19 in a Collaborative Care Model: A Cross-Sectional Study on Self-Management, Information, and Medication Impact by Beatriz Santos, Maeva Rousseau, Ralitza Gauthier, Alexandra Calmy and Marie P. Schneider in Journal of Patient Experience
