# Supplementary material for: Patient Experiences With Nirmatrelvir/Ritonavir for COVID-19 in a Collaborative Care Model: A Cross-Sectional Study on Self-Management, Information, and Medication Impact
Source: J Patient Exp. 2025 May 14;12:23743735251342126. doi: 10.1177/23743735251342126 (PMC12078955; doi:10.1177/23743735251342126)
Supplement: sj-docx-1-jpx-10.1177_23743735251342126 - Supplemental material for Patient Experiences With Nirmatrelvir/Ritonavir for COVID-19 in a Collaborative Care Model: A Cross-Sectional Study on Self-Management, Information, and Medication Impact [file sj-docx-1-jpx-10.1177_23743735251342126.docx]

Figure 2: Participants perceived quality of information provided on nirmatrelvir/r by physicians and pharmacists (1=very poor; 10= excellent)
