# Supplementary material for: Patient Experiences With Nirmatrelvir/Ritonavir for COVID-19 in a Collaborative Care Model: A Cross-Sectional Study on Self-Management, Information, and Medication Impact
Source: J Patient Exp. 2025 May 14;12:23743735251342126. doi: 10.1177/23743735251342126 (PMC12078955; doi:10.1177/23743735251342126)
Supplement: sj-docx-2-jpx-10.1177_23743735251342126 - Supplemental material for Patient Experiences With Nirmatrelvir/Ritonavir for COVID-19 in a Collaborative Care Model: A Cross-Sectional Study on Self-Management, Information, and Medication Impact [file sj-docx-2-jpx-10.1177_23743735251342126.docx]

Paxlovid_remise

Please complete the survey below.

Thank you!

# Part 1: to be completed by the research team BEFORE the call

Questionnaire completed by:

Date of call

Date of Paxlovid dispensing

What is the patient's year of birth?

What is the patient's gender? Female


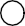

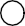

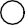


Men Other

# I'd like to start by asking you a few general questions:

Generally speaking, how would you rate your Very good


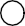

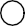

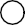

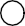

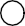


health 7 days ago (at the start of ttt Good

by Paxlovid) ? Average

Poor

Very poor

Generally speaking, how would you rate your Very good


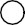

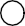

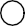

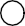

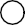


health TODAY? Good

Average

Poor

Very Poor

What medications do you usually take? Please specify: dosage, frequency, since when:

"Medicaiton 1 / 1-0-1-0 / approx 5 years"

# The next questions concern PAXLOVID

Who prescribed Paxlovid? General practitioner Specialist


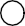

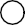

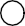

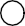

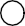


Medical team during my hospitalization Emergency service

Other

Please specify:

Did you pick up the Paxlovid in person or In person


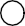

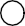

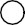

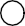

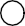


did you ask someone else to A member of my family

collect it for you? A friend

Home care service (ex Imad) Other

Other: please specify:

How did you take Paxlovid?

- Usual regimen: 3 tablets in the morning and 3 tablets in the evening, describe how you took them:

no. of tablets,12-hour interval for 5 consecutive days

-
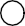
 Modified regimen: specify

Modified regiment: How did you take your treatment? (describe number of tablets / time taken / duration of treatment)

Did you have to make any changes to the No

intake recommendations made by the Lower the dose

doctor and/or pharmacist? (e.g. Reduce the frequency

3-0-0) at another time of day, reduce Increase the frequency

increase dose, stop early Shorten dosing times (< 12h)

treatment or skipping a dose?) Spacing out dosing times (>12h) Other: specify

Please specify

Can you tell me what led you to adapt Because I felt better because I felt unwell / less well (COVID symptoms, side effects, etc.).


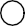

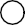

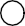


I had fears and/or doubts about the medication (safety, efficacy...)


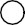
 Because I left home and forgot my medication


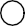
 Other

Please specify

Did someone recommend these changes to you? Who? doctor (check all that apply) pharmacist


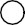

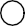

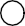

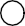

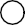

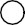


Close Nurse

Internet / social networking Person

Other

Other: Please specify

# The next questions concern information received about Paxlovid

When you left the pharmacy, did you feel you had all the information you needed about PAXLOVID? (on a scale of 1 to 10)


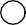
 1
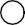
 2
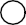
 3
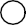
 4
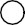
 5
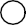
 6
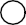
 7
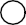
 8
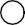
 9
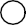
 10

Based on this answer, what On the dose to be taken

information you may have missed? on dosing schedules

On interactions with other medications

On the beneficial effect of the medication

On the medication's potential risks

On its mode of action

Other: please specify

Other: What kind of information did you miss?

Have you found the missing information elsewhere?

No

On the Internet / social networks Via friends / relatives

Via the family

Through my alternative medicine contact Discussing it again with my pharmacist discussion with my doctor

By discussing with another healthcare professional In the package insert

Other

When you left the pharmacy, did you feel you had all the information you needed about your usual medications? (on a scale of 1 to 10)


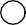
 1
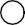
 2
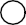
 3
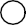
 4
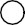
 5
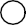
 6
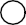
 7
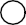
 8
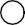
 9
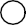
 10

Based on this answer, On the dose to be taken

Did you miss any information? About pick-up times

Interactions with Paxlovid Temporary discontinuation of one or more medications On medications to be continued without any modification

On when to resume my usual medication Other: specify None

Please specify

Have you found the missing information elsewhere?

No

On the Internet / social networks Via friends / relatives

Via the family

Via my alternative medicine contact Discussing it again with my pharmacist

In discussion with my doctor

By talking to another healthcare professional Other

Other: please specify

On a scale of 1 (very poor) to 10 (excellent), how would you describe the quality of the information you received from your doctor?


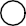
 1
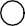
 2
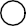
 3
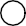
 4
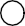
 5
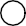
 6
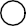
 7
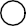
 8
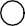
 9
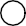
 10

On a scale of 1 (very poor) to 10 (excellent), how would you describe the quality of the information you received from your pharmacist?


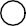
 1
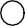
 2
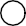
 3
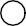
 4
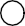
 5
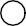
 6
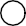
 7
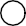
 8
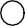
 9
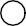
 10

# We now come to the last part of this questionnaire, which concerns the impact of taking Paxlovid on your usual medications.

How has treatment with Paxlovid affected your other medications?

Please specify:

It hasn't affected my medication, I haven't changed anything.

I have stopped one/several medications I have reduced the dose of one/several medications

I have increased the dose of one or more medications

Other

Which medications have been adjusted?

For how many days? 1


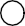

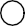

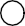

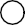

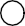

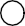

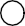

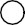

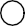

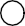


2

3

4

5

6

7

8

9

10 or more

Who recommended these adaptations? Doctor Pharmacist Friends/famiy


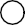

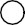

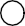

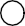

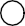

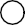


Internet / social networks

I have modified it on my own Other

Other: please specify

Have you taken your usual medication at the usual dosage?

If so, for how many days?

If not, when do you plan to take them back?

Can you tell me more? Items to add

Yes, I have resumed all my medication at the usual dosage.

I have resumed some of my usual medications (please specify)

Yes, I have resumed my medication with modifications/adaptations (please specify) No, I haven't taken my medication yet.

1


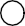

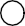

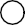

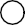

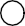

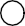

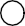


2

3

4

5

6

7 or more

I don't know

I don't think I'll take them back in the next 3 days In the next 7 days In the next 10 days

# THANK YOU FOR YOUR TIME AND COOPERATION.

Call time (min)
