# Supplementary figures and images for: Patient Experiences With Nirmatrelvir/Ritonavir for COVID-19 in a Collaborative Care Model: A Cross-Sectional Study on Self-Management, Information, and Medication Impact
Source: J Patient Exp. 2025 May 14;12:23743735251342126. doi: 10.1177/23743735251342126 (PMC12078955; doi:10.1177/23743735251342126)

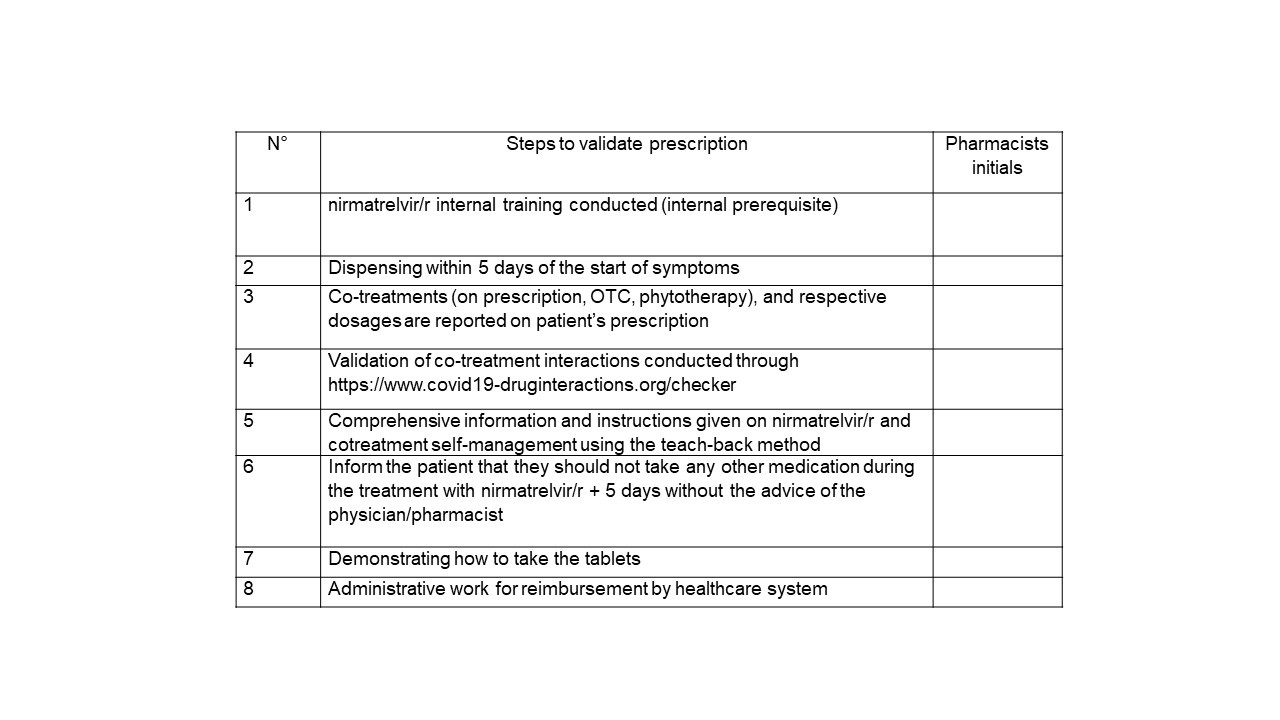


Appendix 1: Local checklist for dispensing of nirmatrelvir/r

Supplement: sj-docx-3-jpx-10.1177_23743735251342126 - Supplemental material for Patient Experiences With Nirmatrelvir/Ritonavir for COVID-19 in a Collaborative Care Model: A Cross-Sectional Study on Self-Management, Information, and Medication Impact [file sj-docx-3-jpx-10.1177_23743735251342126.docx]

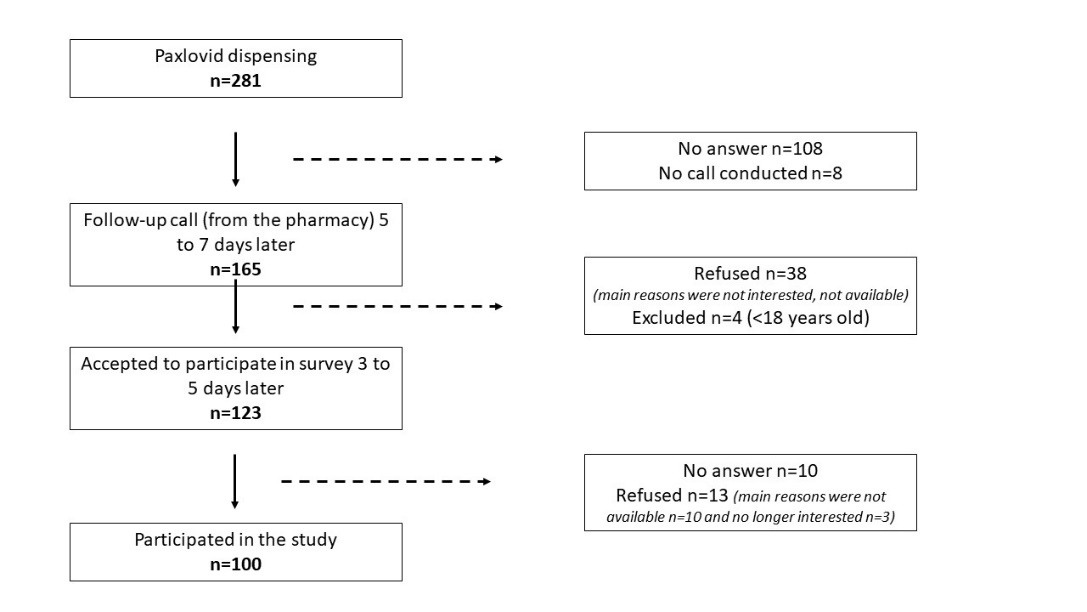


Figure 1: Flowchart of participants inclusion

Supplement: sj-docx-4-jpx-10.1177_23743735251342126 - Supplemental material for Patient Experiences With Nirmatrelvir/Ritonavir for COVID-19 in a Collaborative Care Model: A Cross-Sectional Study on Self-Management, Information, and Medication Impact [file sj-docx-4-jpx-10.1177_23743735251342126.docx]
